# Antimicrobial and Antibiofilm Potential of *Flourensia retinophylla* against *Staphylococcus aureus*

**DOI:** 10.3390/plants13121671

**Published:** 2024-06-17

**Authors:** Minerva Edith Beltrán-Martínez, Melvin Roberto Tapia-Rodríguez, Jesús Fernando Ayala-Zavala, Agustín Gómez-Álvarez, Ramon Enrique Robles-Zepeda, Heriberto Torres-Moreno, Diana Jasso de Rodríguez, Julio César López-Romero

**Affiliations:** 1 Coordinación de Tecnología de Alimentos de Origen Vegetal, Centro de Investigación en Alimentación y Desarrollo, A.C. Carretera Gustavo Astiazarán Rosas No. 46, Colonia la Victoria, Hermosillo 83304, Mexico; mbeltran122@estudiantes.ciad.mx (M.E.B.-M.); jayala@ciad.mx (J.F.A.-Z.); 2 Departamento de Biotecnología y Ciencias Alimentarias, Instituto Tecnológico de Sonora, 5 de Febrero 818 sur, Col. Centro, Ciudad Obregón 85000, Mexico; melvin.tapia14987@potros.itson.edu.mx; 3 Departamento de Ingeniería Química y Metalurgia, Universidad de Sonora, Hermosillo 83000, Mexico; agustin.gomez@unison.mx; 4 Departamento de Ciencias Químico Biológicas, Universidad de Sonora, Hermosillo 83000, Mexico; robles.zepeda@unison.mx; 5 Departamento de Ciencias Químico Biológicas y Agropecuarias, Universidad de Sonora, Caborca 83600, Mexico; heriberto.torres@unison.mx; 6 Universidad Autónoma Agraria Antonio Narro, Saltillo 25315, Mexico

**Keywords:** natural products, *F. retinophylla*, *S. aureus*, planktonic cells, biofilms

## Abstract

*Staphylococcus aureus* is a Gram-positive bacteria with the greatest impact in the clinical area, due to the high rate of infections and deaths reaching every year. A previous scenario is associated with the bacteria’s ability to develop resistance against conventional antibiotic therapies as well as biofilm formation. The above situation exhibits the necessity to reach new effective strategies against this pathogen. *Flourensia retinophylla* is a medicinal plant commonly used for bacterial infections treatments and has demonstrated antimicrobial effect, although its effect against *S. aureus* and bacterial biofilms has not been investigated. The purpose of this work was to analyze the antimicrobial and antibiofilm potential of *F. retinophylla* against *S. aureus*. The antimicrobial effect was determined using an ethanolic extract of *F. retinophylla*. The surface charge of the bacterial membrane, the K^+^ leakage and the effect on motility were determined. The ability to prevent and remove bacterial biofilms was analyzed in terms of bacterial biomass, metabolic activity and viability. The results showed that *F. retinophylla* presents inhibitory (MIC: 250 µg/mL) and bactericidal (MBC: 500 µg/mL) activity against *S. aureus*. The MIC extract increased the bacterial surface charge by 1.4 times and the K^+^ concentration in the extracellular medium by 60%. The MIC extract inhibited the motility process by 100%, 61% and 40% after 24, 48 and 72 h, respectively. The MIC extract prevented the formation of biofilms by more than 80% in terms of biomass production and metabolic activity. An extract at 10 × MIC reduced the metabolic activity by 82% and the viability by ≈50% in preformed biofilms. The results suggest that *F. retinophylla* affects *S. areus* membrane and the process of biofilm formation and removal. This effect could set a precedent to use this plant as alternative for antimicrobial and disinfectant therapies to control infections caused by this pathogen. In addition, this shrub could be considered for carrying out a purification process in order to identify the compounds responsible for the antimicrobial and antibiofilm effect.

## 1. Introduction

Bacterial infections constitute a public health problem worldwide due to the substantial increase in the number of cases reported in recent years [1]. This has been associated with the ability of microorganisms to resist conventional antibiotic therapies used in clinical practice, which has resulted in increased morbidity and mortality rates [2]. In this regard, the World Health Organization (WHO) estimates that bacterial infections will be the leading cause of death worldwide by 2050, causing more than 10 million deaths per year [3].

One of the most relevant Gram-positive bacteria in public health is *Staphylococcus aureus*, considered by the WHO as a high priority microorganism due to its antibiotic resistance [4]. Furthermore, *S. aureus* has been classified within the ESKAPE bacteria because of its high virulence and resistance to antibiotics [5]. In addition, this microorganism is highly associated with community and hospital infections, with more than 110,500 deaths estimated in 2019 [6]. Some pathologies associated with this bacteria include skin infections, soft tissue infections of the lower respiratory tract, bacteremia, osteomyelitis and endocarditis, which can become chronic, persistent and a cause of death [7]. Additionally, this bacteria is one of the main sources of contamination of medical devices and instrumentation [8]. This is mainly associated with the ability of *S. aureus* to adhere to different surfaces and form biofilms [9].

Biofilms are defined as communities of microorganisms that grow embedded in a layer of exopolysaccharides, which are mainly composed of polysaccharides, proteins, genetic material and lipids [10]. These communities are formed in a four-stage process: adhesion, synthesis of extracellular matrix, formation and maturation of the biofilm, and detachment of bacterial cells [11]. These structures confer to the bacterial community resistance against antibiotics and the immune system, which makes treatment difficult and causes persistent infections, since they act as a continuous focus of infection [12].

As shown above, the current strategies for the control of *S. aureus* are not effective, demonstrating the claim to develop new effective alternatives for the control of *S. aureus* infections and their biofilms to reduce their impact on health. In this sense, plants could represent a feasible strategy, used in traditional medicine against different health conditions, including bacterial infections, where it is estimated around 80% of the world population uses plants as primary treatment for different health conditions [13]. *Flourensia retinophylla* S.F. blake, a plant known as “yerba de mula”, is widely distributed in Coahuila, Mexico and it is used in traditional medicine against infections [14]. Recent research has provided information about different biological activities such as antimicrobial; however, its antimicrobial effect against *S. aureus* has not been reported and its antibiofilm effect has not been studied [14,15,16]. The biological potential of this plant is associated with the presence of bioactive compounds, especially phenolic compounds and terpenes [14,15].

Based on the above, the objective of this work was to determine the effect of *F. retinophylla* on planktonic cells of *S. aureus* and on the prevention and removal of bacterial biofilms of such pathogens.

## 2. Results and Discussions

In recent years, there has been a global increase in infections caused by bacteria. Those caused by antibiotic-resistant bacteria are of the greatest concern since the existing pharmacological treatments have decreased or even lost their efficiency [17]. Given this scenario, the need arises to explore new therapies capable of combating or reducing the incidence of infectious diseases associated with resistant pathogens. In this context, traditional medicine becomes more relevant since currently around 80% of the population uses it as primary treatment [13]. In addition, a considerable number of drugs have been derived from natural sources and plants are a notable source due to the presence of various secondary metabolites that have been reported to confer antimicrobial activity [18]. Among the plants used by ethnic groups, *F. retinophylla* stands out. Despite limited previous research on this plant, there has been interest in examining its antimicrobial and antibiofilm effects. This study reports for the first time the antimicrobial and antibiofilm activity of *F. retinophylla* against *S. aureus*, one of the main microorganisms causing nosocomial infections.

Antimicrobial assessment showed that the ethanolic extract of *F. retinophylla* effectively inhibited the growth of *S. aureus* with a minimum inhibitory concentration (MIC) of 250 µg/mL and a minimum bactericidal concentration (MBC) of 500 µg/mL. According to Simoes et al. [19], an MIC equal to or lower than 1000 µg/mL suggests that the natural source is a potential antimicrobial agent, thus revealing a promising role of this plant as bactericidal. Additionally, regarding edible plant extracts or their parts, it is estimated that they are very active if they show MIC values <100 µg/mL, significantly active if 100 ≤ MIC ≤ 512 µg/mL, moderately active if 512 ≤ MIC ≤ 2048 µg/mL and not very active if the MIC > 2048 µg/mL [20]. The biological potential of this plant could be associated with the nature of the chemical compounds present in the plant. Previously, our work group demonstrated the presence of bioactive compounds such as flavonoids, phenolic acids, and terpenes in this extract [14,15], which have been shown to exhibit antimicrobial effects against *S. aureus*, such as apigenin (MIC: 31.25 µg/mL), quercetin (MIC: 300 µg/mL), ellagic acid (MIC: 128 µg/mL) and phytol (MIC: >1000 µg/mL) [21,22,23,24].

**Figure 1:**
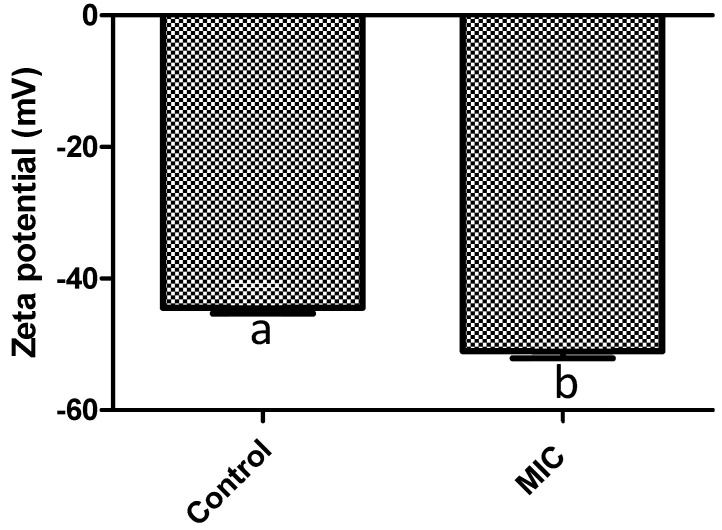
Zeta potential values (mV) of *S. aureus* after 1 h of exposure to *F. retinophylla* MIC ethanolic extract. Data are presented as mean ± standard deviation. ^a-b^ Mean with different letter are different (*p* < 0.05).

On the other hand, the antimicrobial mode of action of *F. retinophylla* has not been previously reported. Thus, in order to know the mode of action associated with the antimicrobial effect of *F. retinophylla* extract, we evaluated its effect on the surface charge of *S. aureus* (Figure 1). We observed an increase of 1.4 times the surface charge of the bacteria treated with *F. retinophylla* MIC extract compared to the vehicle control (*p* < 0.05).

It is well known that the bacterial cell membrane has a negative cell surface charge due to its constituents [25]. Hence, modifications in the surface charge may indicate modifications in the bacterial membrane integrity. The *F. retinophylla* extract increased the surface charge of *S. aureus*, suggesting that the bioactive compounds present in the extract interact with the bacterial cell membrane. This charge change could be related to the ability of the plant compounds to modulate membrane potential, similar to other studies that have shown that phenolic compounds and terpenes have the ability to interact with the bacterial cell wall and membrane of *S. aureus*, altering the charge of the bacterial cell surface [26,27,28].

**Figure 2:**
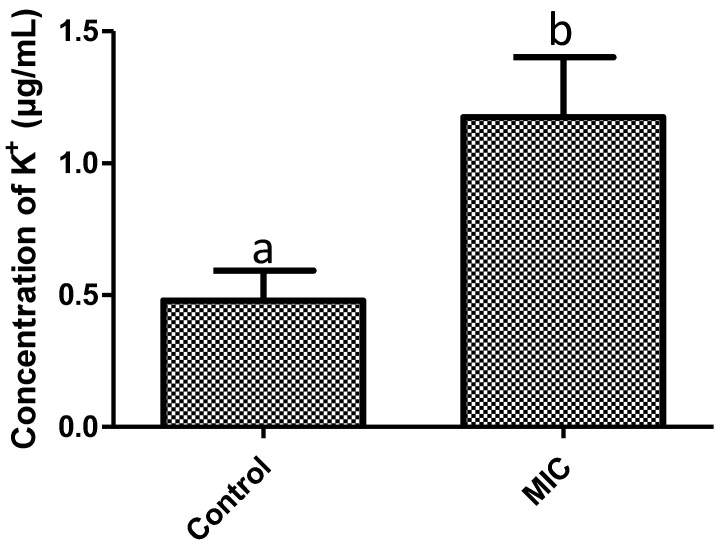
Concentration of K^+^ (µg/mL) in solution of *S. aureus* after 1 h of exposure to *F. retinophylla* MIC ethanolic extract. Data are presented as mean ± standard deviation. ^a-b^ Mean with different letter are different (*p* < 0.05).

In the study of the mode of action of *F. retinophylla* against *S. aureus*, K^+^ leak was assessed, which identifies changes in the permeability of the bacterial cell membrane. After 1 h of treatment with *F. retinophylla,* MIC extract produced a 60% increase in the K^+^ concentration in the extracellular medium compared to the control group (*p* < 0.05) (Figure 2).

The cytoplasmic membrane is crucial to maintain the homeostasis of bacterial cells, regulating the entry and exit of intracellular components [29]. K^+^ is one of the main constituents of the bacterial cytoplasm because it plays an important role in the metabolic processes [30]. The high concentration of K^+^ in the extracellular medium found in this study suggests an alteration of the cytoplasmic membrane which may cause membrane disruption and subsequent leakage of intracellular components leading to bacterial cell death [31]. This effect may be associated with the bioactive compounds present in the *F. retinophylla* extract, since phenolic compounds and terpenes were shown to induce an alteration in the cytoplasmic membrane of *S. aureus*, inducing leakage of intracellular components including K^+^ [24,27,28].

**Figure 3:**
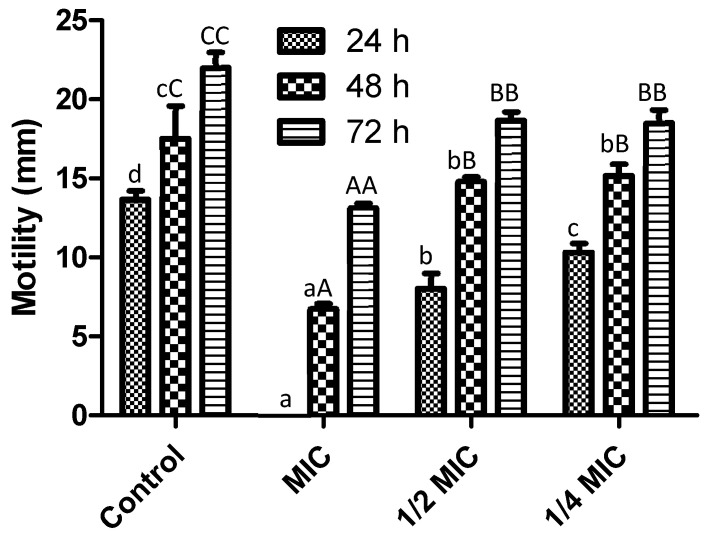
Motility (mm) of *S. aureus* after 72 h of exposure to different *F. retinophylla* ethanolic extract concentration. Data are presented as mean ± standard deviation. ^a–d: 24 h; aA–cC: 48 h; AA–CC: 72 h^ Mean with different letter between the different time of incubation (24, 48 and 72 h) are different (*p* < 0.05).

On the other hand, bacterial motility plays an essential role in the virulence and pathogenicity of bacteria. We analyzed the effect of the *F. retinophylla* extract on the motility of *S. aureus* (Figure 3). Our results showed that the extract inhibited the motility of *S. aureus* at all concentrations tested. MIC was the most active concentration (*p* < 0.05), showing a dose–response effect and inhibiting motility by 100%, 61% and 40% after 24 h, 48 h and 72 h, respectively. Furthermore, ½ MIC (between 41–15% at 24–72 h, respectively) and ¼ MIC (between 24–15% at 24–72 h, respectively) also decreased pathogen motility (*p* < 0.05).

Results reveals that MIC concentration inhibits the motility process and subsequently increases over time, similar with the other evaluated concentrations. In this sense, it was demonstrated that MIC or lower concentrations of plant extracts could be able to inhibit the *S. aureus* motility process after 24 h [32]. Also, Vazquez-Armenta et al. [33] observed that grape stem extract inhibited the motility process in *L. monocytogenes* after 24 h; however, this process gradually increased after 48 and 72 h. The bacterial motility mechanism is not elucidated at all; however, it is important to highlight that motility correlates with the cellular state of the microorganism [34]. In this sense, MIC did not inhibit the entire bacterial viability; therefore, it could be suggested the presence of a minimum count of viable cells that subsequently managed to adapt and develop under stress conditions, such as antimicrobial treatments. Otherwise, the motility is vital for the adaptation, survival, colonization, biofilm development and virulence of bacteria [35]. In particular, *S. aureus* displays a motility process called spreading [36]. This process is related to two factors: the agr quorum sensing system and the production of phenol-soluble modulin surfactants. These molecules have lytic activity against leukocytes and erythrocytes, cause pro-inflammatory effects and interfere with the development of biofilms [36,37]. The results of this study showed that the *F. retinophylla* extract inhibited the motility process of *S. aureus* which could be related to the bioactive compounds present in the extract. For example, it was previously shown that phenolic compounds (luteolin, -hydroxyemodin and 3-hydroxybenzoic acid) and terpenes (eugenol and (+)-nootkatone) affect the *quorum sensing* system of *S. aureus* and its agr system [38,39,40,41,42], inhibiting the transcriptional units RNAII and RNAIII, which are involved in the production of virulence factors such as phenol-soluble modulin [38,43,44]. Furthermore, the agr *quorum sensing* system of *S. aureus* is associated with the bacterial membrane [45]. In this study, we showed that the *F. microphylla* extract alters the membrane integrity of *S. aureus*. Based on this, we hypothesize that: (i) the *F. retinophylla* extract could affect the agr *quorum sensing* system of *S. aureus*, inhibiting the production of phenol-soluble modulin, and (ii) the alteration of the bacterial membrane could affect the detection of the central *quorum sensing* system in *S. aureus*.

This study is the first one to report the antimicrobial effect of *F. retinophylla* against *S. aureus*, one of the most relevant Gram-positive pathogens in the clinic. We demonstrated that the effect against planktonic cells of *S. aureus* is related to the ability of the extract to induce damage in the bacterial cell wall and membrane, causing irreversible damage and subsequent cell death. Furthermore, we revealed that *F. retinophylla* significantly reduces motility on *S. aureus,* which may diminish bacterial pathogenicity.

**Figure 4:**
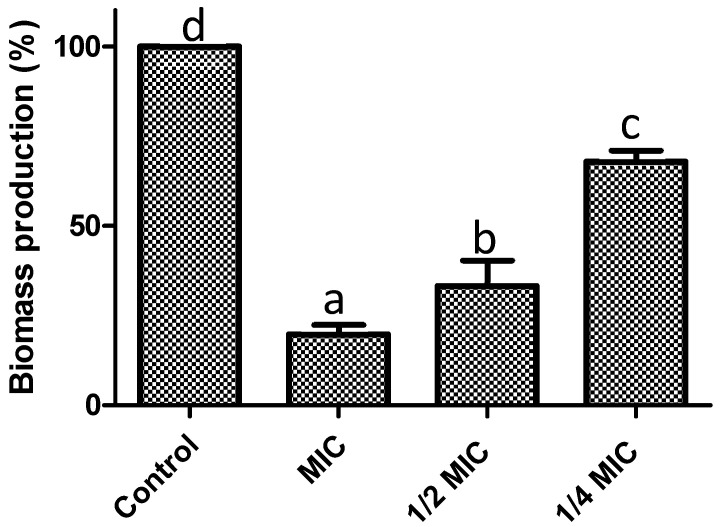
Preventive effect on biofilm formation of *F. retinophylla* ethanolic extract at MIC, ½ MIC and ¼ MIC on biomass production of *S. aureus*. Data are presented as mean ± standard deviation. ^a–d^ Mean with different letter are different (*p* < 0.05).

**Figure 5:**
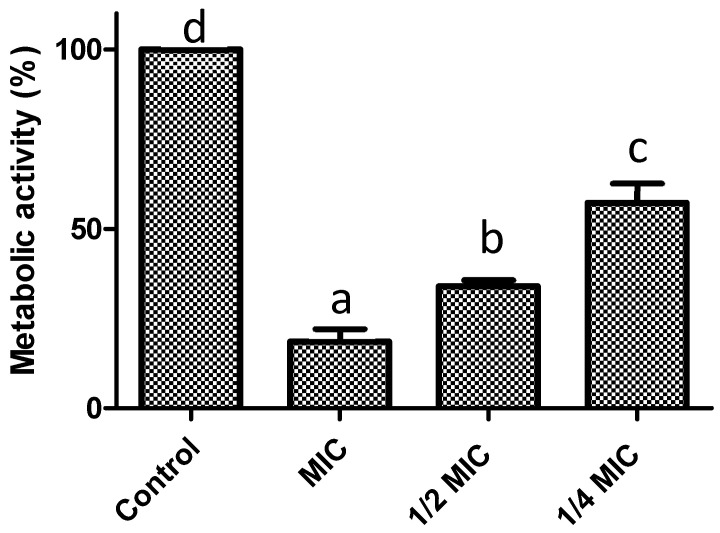
Preventive effect on biofilm formation of *F. retinophylla* ethanolic extract at MIC, ½ MIC and ¼ MIC on metabolic activity of *S. aureus*. Data are presented as mean ± standard deviation. ^a–d^ Mean with different letter are different (*p* < 0.05).

Another important factor in the pathogenesis of *S. aureus* is its ability to form biofilms. In this sense, we assessed the ability of *F. retinophylla* to inhibit the formation of *S. aureus* biofilms. The extract significantly inhibited biomass formation (*p* < 0.05) by 80% at MIC, by 67% at 1/2 MIC and by 32% at 1/4 MIC (Figure 4). The extract also reduced the metabolic activity of the bacterial cells of the biofilms (*p* < 0.05), being most effective at MIC with a reduction of 82% (Figure 5). Concentrations of 1/2 MIC and 1/4 MIC also reduced (*p* < 0.05) the metabolic activity of biofilms by 66% and 43%, respectively.

The first step for the formation of biofilms is the reversible attachment of cells to the surface to be colonized [46]. This process is associated with motility and with the proteins and genes that regulate adhesion [47]. Here we showed that *F. retinophylla* significantly reduces biofilm formation of *S. aureus*. This effect could be initially associated with damage to the planktonic cells, on top of damage to the membrane where the proteins that regulate adhesion are located. Furthermore, the extract demonstrated to reduce the motility process of *S. aureus* and possibly affect the quorum sensing system. The bioactive compounds present in the extracts, such as flavonoids, demonstrated interaction with proteins that regulate adhesion and biofilm formation such as AtIE, Bap, IcaA, SarA, SasG [48,49]. Other studies have shown that phenolic compounds and terpenes exhibit the ability to affect the expression of genes associated with biofilm formation such as *sarA*, *agrA*, *icaA*, *spa*, *sdrD*, *hld*, *cap5B* and *cap5C* [50,51]. These studies also demonstrated that flavonoids and terpenes exhibit the ability to inhibit biofilm formation of *S. aureus*. Based on these findings, we suggest that the previously reported bioactive compounds present in the *F. retinophylla* extract could interact with proteins involved in the regulation of genes that control adhesion and biofilm formation of *S. aureus*, resulting in a high inhibition of biofilm formation of this pathogen.

**Figure 6:**
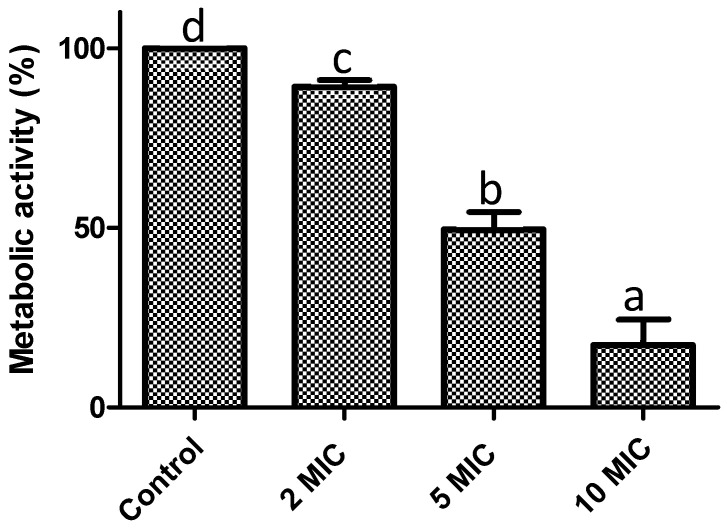
Effect of *F. retinophylla* ethanolic extract against pre-established biofilms (24 h) of *S. aureus* in terms of metabolic activity after 1 h of exposure to different concentration of *F. retinophylla* ethanolic extract. Data are presented as mean ± standard deviation. ^a–d^ Mean with different letter are different (*p* < 0.05).

**Figure 7:**
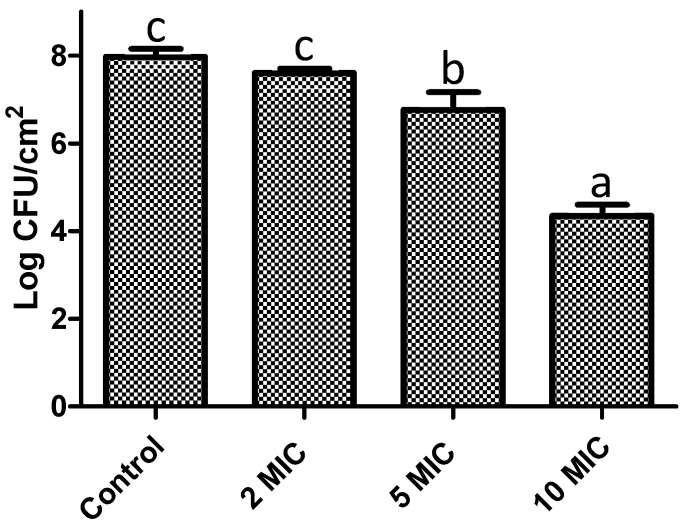
Effect of *F. retinophylla* ethanolic extract against pre-established biofilms (24 h) of *S. aureus* in terms of remaining viable biofilm cells after 1 h of exposure to different concentration of *F. retinophylla* ethanolic extract. Data are presented as mean ± standard deviation. ^a–c^ Mean with different letter are different (*p* < 0.05).

Another crucial factor is biofilms maturation, as it becomes highly complicated to control or eradicate it at this stage. In this sense, we assessed the ability of *F. retinophylla* to remove 24-h-preformed biofilms of *S. aureus* after being exposed to the extract for 1 h (Figure 6). Initially, the capacity of the extract to affect the metabolic activity of the preformed biofilms was evaluated. A concentration-dependent effect was observed (*p* < 0.05) since the highest evaluated concentration (10 MIC) showed the greatest reduction (83%), followed by the concentration of 5 MIC (50%) and 2 MIC (11%) after 1 h of contact. Then, the ability of *F. retinophylla* to affect the viability of the cells contained in the 24-h-preformed biofilms was determined by exposed them in contact for 1 h (Figure 7). Again, a similar effect was observed, where the concentration of 10 MIC decreased the viability of the biofilm cells by ∼50% (*p* < 0.05), followed by the extract at 5 MIC with a 15% reduction (*p* < 0.05); however, the extract at 2 MIC did not affect bacterial viability.

These results demonstrate that *F. retinophylla* has a high capacity to remove preformed biofilms of *S. aureus* after 1 h of exposition. This effect may be associated with the compounds previously reported to be present in the extract. Phenolic compounds and terpenes have been shown to alter bacterial biofilms by modifying the exopolysaccharide structure, i.e., reducing the concentration of polysaccharides, proteins and DNA of these structures [52,53,54,55]. Furthermore, microscopy studies have demonstrated that these bioactive compounds can degrade bacterial biofilms of *S. aureus* by being in contact [56,57]. This suggests that the bioactive compounds of the *F. retinophylla* extract may interact with the exopolysaccharide structure of *S. aureus* and damage its architecture, possibly allowing the passage of bioactive compounds that may interact with the cells and modify their metabolism and reduce their viability, resulting in bacterial cell death. In turn, it could be suggested that the bioactive compounds could cross the biofilms by passive diffusion and interact with the bacterial cells on the inside, producing a loss of their viability.

This work may be of interest in the clinic since a large number of infections caused by *S. aureus* and more than 80% of nosocomial infections are associated with bacterial biofilms [58,59]. In addition, these structures represent a serious challenge for the public health, as there are currently no effective strategies that can eradicate them after they form in the human organism [60]. It is worth mentioning that the exopolysaccharide structures provide the bacterial community with the ability to evade the effect of immune system cells, such as neutrophils, macrophages and antibodies [61]. It has also been observed that biofilms confer resistance against antimicrobials used in clinical practice and that it can be between 10 to 100 times higher compared to planktonic cells [62]. Moreover, once biofilms reach maturity, bacterial cells begin to detach and may colonize other biological surfaces, generating what is known as microbial metastasis [63]. These behaviors cause that the development of biofilms in the organisms gives rise to chronic, persistent infections and possible spread that in severe cases can lead to the death of the patient [64].

The results found in this study report unpublished and original data. To our knowledge, this is the first study to report the antibiofilm effect of *F. retinophylla*, specifically against *S. aureus* which constitutes a challenge in public health due to the antibiotic resistance that it has gained in recent years. Based on this work, we consider that *F. retinophylla* represents a possible source for the development of antimicrobial and/or disinfectant agents, which may be applied in humans for the treatment of bacterial infections caused by *S. aureus*. However, it is necessary to research and guarantee the safety of the use of this natural source.

## 3. Materials and Methods

### 3.1. Plant Material Obtention

*F. retinophylla* S.F. Blake was collected in Sierra Paila, Coahuila, Mexico during September 2021. The plant was identified at the Herbarium of the Universidad Autónoma Agraria Antonio Narro by Dr. José Ángel Villarreal Quintanilla (voucher 82956). The plants were transported to the Phytochemistry Laboratory of the Universidad Autónoma Agraria Antonio Narro, where they were dried in an oven (60 °C for 48 h) and grounded (2 mm sieve).

### 3.2. Preparation of the Extract

The extraction process was carried out using a Soxhlet method with ethanol as the extraction solvent. A total of 14 g of ground *F. retinophylla* leaves was extracted with 200 mL of ethanol for 72 h. After, the solvent was removed using a rotary evaporator. The residual solvent was further eliminated in an oven (50 °C for 24 h). The obtained dry extract was stored frozen at −20 °C until use.

All methods used in this study were performed in triplicate.

### 3.3. Bacterial Strain

The microorganism used in this research was *Staphylococcus aureus* ATCC 25923. The bacterial strain was preserved at −80 °C in cryovials containing Mueller–Hinton broth and glycerol (30% *v*/*v*). Before use, the bacterial strain was activated (37 °C for 24 h) on Mueller–Hinton broth.

### 3.4. Antimicrobial Evaluation

The antimicrobial effect of *F. retinophylla* extract was carried out using a reported method by Velazquez et al. [65]. Fresh overnight growth bacteria (16–18 h at 37 °C) in Mueller–Hinton broth was adjusted at 0.5 McFarland (1 × 10^8^ colony forming units (CFU)/mL). Afterward, 15 µL of the adjusted bacteria were inoculated into 96-well polystyrene microplate (Costar, Corning, NY, USA) containing 200 µL of different extract concentrations (62.5–1000 µg/mL). *F. retinophylla* extract was dissolved in dimethyl sulfoxide (DMSO) and diluted in Mueller–Hinton broth. The concentration of DMSO was less than 2% (weight/volume: *w*/*v*). DMSO (2%, *w*/*v*) and gentamicin (12 µg/mL) were utilized as controls. The microplates were incubated at 37 °C for 24 h. Afterward, the absorbance was read at 620 nm in a microplate reader (Multiskan Fc, Thermo Scientific, Bohemia, NY, USA). The minimal inhibitory concentration (MIC) was defined as the lowest concentration that inhibited bacterial growth. After, 10 µL of equal or lower concentration than MIC was inoculated onto plate count agar and incubated at 37 °C for 24 h. The concentration that showed no growth was defined as the minimal bactericidal concentration (MBC).

### 3.5. Surface Charge Determination

Fresh overnight culture (16–18 h) was adjusted to a cell density of 1 × 10^6^ CFU/mL. The adjusted bacteria were brought into contact with the MIC extract and incubated for 1 h at 37 °C. The mixture was centrifugated twice (6000× *g* for 10 min) and resuspended in sterile water. Finally, bacteria surface charge was calculated by zeta potential using Zeta-sizer Nano-ZS90 (Malvern Instruments Ltd., Worcestershire, UK), with deionized water utilized as a diluent [27].

### 3.6. Potassium (K^+^) Leakage

The potassium leakage was analyzed using flame emission and atomic absorption spectroscopy for K+ tritation in *S. aureus* suspension incorporated with MIC extract. The mixture was kept in contact for 1 h and was filtered (sterile membrane filter size of 0.22 µM). The sample was analyzed by atomic absorption spectroscopy, using a Perking-Elmer atomic absorption equipment AAnalyst 400 (Perkin Elmer, Shelton, CT, USA) [27].

### 3.7. Motility Assay

Bacterial motility was evaluated following the method described by Abreu et al. [66] with some modifications. Fresh overnight culture (16–18 h) was adjusted at 1 × 10^8^ CFU/mL. After that, 10 µL of the bacterial inoculum was transferred in the center of a Petri dish with 0.3% agar in the presence of MIC, ½ MIC, and ¼ MIC of *F. retinophylla* extract. The extract was incorporated into the medium at 45 °C. DMSO was used as a control. The inoculated Petri dish was stored at 30 °C and measured at 24, 48 and 72 h.

### 3.8. Biofilm Inhibition Effect—Inhibitory Effect of Initial Bacterial Cells Attachment

The effect of *F. retinophylla* extract to prevent the biofilm formation of *S. aureus* was performed using the method described by Bazargani and Rohloff [67]. A total of 100 µL of MIC, ½ MIC and ¼ MIC of *F. retinophylla* extract were incorporated into each well of a 96-well polystyrene microplate (Costar, Corning, NY, USA). DMSO was used as a control. Subsequently, 100 µL of inoculum (1 × 10^6^ CFU/mL) was added to the wells. The microplates were incubated for 8 h at 37 °C. Subsequently, the biomass production and metabolic activity were quantified.

Biomass production: the content of each well was washed with 200 µL of saline solution (0.85% *w*/*v*) and fixed with 200 µL of methanol for 15 min. Subsequently, the microplates were air-dried and stained with 200 µL of crystal violet (CV, 1%, volume/volume, *v*/*v*). The CV was removed, and 200 µL of glacial acetic acid (33%, *v*/*v*) was added to the wells. Finally, a microplate reader (Multiskan Fc, Thermo Scientific) was used to measure the absorbance at 590 nm. Results were expressed as a percentage of biomass production [62].

Metabolic activity: the content of each well was washed with saline solution (0.85% *w*/*v*). XTT was dissolved in saline solution (0.85%, *w*/*v*) to obtain a 1 mg/mL concentration. Subsequently, the solution was filter-sterilized and stored at −80 °C. Menadione was dissolved in acetone to obtain a 1 mM concentration and sterilized. Each well was then filled with 200 µL of saline solution (0.85%, *w*/*v*), and 27 µL of an XTT (sodium 3′-[1-(phenylaminocarbonyl)-3,4-tetrazolium]-bis(4-methoxy6-nitro) benzene sulfonic acid hydrate)/menadione mixture (relation 12.5:1) was added. The microplates were incubated in darkness at 37 °C for 2–3 h. Absorbance was measured at 490 nm in a microplate reader (Multiskan Fc, Thermo Scientific). Results were represented as a percentage of metabolic activity [67].

### 3.9. Biofilm Control

The effect of *F. retinophylla* extract to remove preformed biofilm of *S. aureus* was evaluated based on the method previously described by Borges et al. [62]. Fresh overnight culture (16–18 h) was adjusted to a cell density of 1 × 10^8^ CFU/mL. Subsequently, 200 µL of inoculum was added to a 96-well polystyrene microplate (Costar, Corning, NY, USA) and incubated for 24 h at 37 °C. After, the content of each well was washed with 200 µL of saline solution (0.85% *w*/*v*). *F. retinophylla* extract was performed at different concentrations (2 × MIC, 5 × MIC, 10 × MIC) and incorporated in each well of a 96-well microplate and incubated for 1 h at 37 °C. DMSO was used as a control. After, the metabolic activity and biofilm viable cells were quantified.

Metabolic activity was analyzed using the method previously described.

Biofilm viable cells: the content of each well was washed with saline solution (0.85% *w*/*v*). Each well was then filled with 200 µL of saline solution (0.85%, *w*/*v*) and scrapped from the microtiter plate using a pipette tip for 1 min. This process was replicated three times. After, serial dilutions (1:10) were performed in saline solution (0.85%, *w*/*v*). Subsequently, 10 µL of each dilution was cultured on plate count agar. The inoculated plates were incubated for 24 h at 37 °C. Finally, the bacterial colonies were quantified. Results were represented as log CFU/cm^2^ [62].

### 3.10. Statistical Analysis

The data were analyzed through ANOVA using the software NCSS, 2007. The analyzed variables in this study were surface charge, potassium leakage, motility, biomass production, metabolic activity and viable count. In all cases, data is presented as mean ± standard deviation. When significance differences were observed between treatments means, a Tukey–Kramer test were carried out. The level of significance in the error was *p* < 0.05.

## 4. Conclusions

This study is the first to showcase the potential antimicrobial and antibiofilm effects of *F. retinophylla* against *S. aureus*. Further research is required to conduct toxicological tests to evaluate the safety of *F. retinophylla*. Future studies should focus on identifying the specific compounds in *F. retinophylla* responsible for its antimicrobial and antibiofilm effects. Additionally, the obtained results open the door for the analysis of the antimicrobial and antibiofilm effect of this plant source against other microorganisms of relevance in the health sector. Finally, the results give additional scientific support to the medicinal use of the analyzed species to treat infections.

## Data Availability

The original contributions presented in the study are included in the article, further inquiries can be directed to the corresponding authors.
